# Patient Perspectives on the Experience of Being Newly Diagnosed with HIV in the Emergency Department/Urgent Care Clinic of a Public Hospital

**DOI:** 10.1371/journal.pone.0074199

**Published:** 2013-08-26

**Authors:** Katerina A. Christopoulos, Amina D. Massey, Andrea M. Lopez, C. Bradley Hare, Mallory O. Johnson, Christopher D. Pilcher, Hegla Fielding, Carol Dawson-Rose

**Affiliations:** 1 HIV/AIDS Division, San Francisco General Hospital, University of California San Francisco, San Francisco, California, United States of America; 2 Center for AIDS Prevention Studies, University of California San Francisco, San Francisco, California, United States of America; Fundacion Huesped, Argentina

## Abstract

We sought to understand patient perceptions of the emergency department/urgent care (ED/UC) HIV diagnosis experience as well as factors that may promote or discourage linkage to HIV care. We conducted in-depth interviews with patients (n=24) whose HIV infection was diagnosed in the ED/UC of a public hospital in San Francisco at least six months prior and who linked to HIV care at the hospital HIV clinic. Key diagnosis experience themes included physical discomfort and limited functionality, presence of comorbid diagnoses, a wide spectrum of HIV risk perception, and feelings of isolation and anxiety. Patients diagnosed with HIV in the ED/UC may not have their desired emotional supports with them, either because they are alone or they are with family members or friends to whom they do not want to immediately disclose. Other patients may have no one they can rely on for immediate support. Nearly all participants described compassionate disclosure of test results by ED/UC providers, although several noted logistical issues that complicated the disclosure experience. Key linkage to care themes included the importance of continuity between the testing site and HIV care, hospital admission as an opportunity for support and HIV education, and thoughtful matching by linkage staff to a primary care provider. ED/UC clinicians and testing programs should be sensitive to the unique roles of sickness, risk perception, and isolation in the ED/UC diagnosis experience, as these things may delay acceptance of HIV diagnosis. The disclosure and linkage to care experience is crucial in forming patient attitudes towards HIV and HIV care, thus staff involved in disclosure and linkage activities should be trained to deliver compassionate, informed, and thoughtful care that bridges HIV testing and treatment sites.

## Introduction

Since the 2006 publication of the Centers for Disease Control and Prevention guidelines recommending HIV screening in all health care settings [1], there has been considerable public health interest in developing the emergency department (ED) as an HIV testing site. As funding opportunities have allowed ED HIV testing to expand, researchers have attempted to determine optimal testing strategies. These studies have focused largely on ascertaining the operational aspects that result in greater test uptake and increased yield of new HIV diagnoses [2]. Though some studies have assessed patient satisfaction with ED HIV testing, most of these investigations have been quantitative or have focused exclusively on individuals who received negative test results [3–5]. Only one study has explicitly assessed satisfaction with the testing experience in those who tested positive for HIV infection [6]. Using a Likert scale survey, researchers in a Boston ED found that 34.5% of participants with reactive test results reported less than optimal satisfaction with the ED HIV testing process. However, the authors acknowledge that it is unclear whether this finding is due to true dissatisfaction with testing or dismay at the positive test result. In addition, although linkage to care rates have been incorporated into ED HIV testing program outcomes, little is known about the components of the ED diagnosis experience that impact subsequent engagement in care. Moreover, EDs may face the challenge of patients leaving before results are available for disclosure, requiring outreach for linkage. A study that looked at factors associated with linkage to care following an ED HIV diagnosis did not include satisfaction with the testing experience as a predictor [7].

Despite many quantitative program evaluations, there is, to our knowledge, no published exploration of the patient experience of being newly diagnosed with HIV in the ED or an urgent care (UC) setting. A qualitative assessment of the ED/UC HIV diagnosis experience can yield meaningful patient-centered insights to help improve the delivery of HIV testing and linkage to care in these locations, especially since qualitative methods allow participants to raise topics of importance. As such, an in-depth exploration of the patient experience constitutes a key part of comprehensive program evaluation [8]. We therefore sought to understand patient perceptions of the ED/UC HIV diagnosis experience, specifically attending to factors that may promote or discourage linkage to care.

## Methods

### Ethics Statement

As part of a larger qualitative study examining the HIV care continuum, we conducted in-depth interviews after obtaining verbal informed consent [9]. The research team chose to use verbal consent because a signed consent form would have been the only link between the study and the participant, constituting the primary source of risk for potential loss of confidentiality. Participants were given an information sheet signed by the study interviewer documenting their verbal consent. The Committee on Human Research at the University of California San Francisco approved all study procedures.

### Study Setting and Population

The county hospital of San Francisco serves an urban population affected by health and socio-economic disparities. The ED HIV testing program at this hospital has been described in detail elsewhere [10], but in brief, testing is diagnostic and targeted; opt-in; and offered by ED clinicians. The hospital laboratory conducts round-the-clock rapid testing on venipuncture specimens and communicates all reactive results to an HIV clinic-based linkage to care team, which assists with disclosure and follow up [11]. As test results are received within two hours of receipt in laboratory, most disclosures to patients happen on-site, though a small number require telephone outreach after patient discharge. Urgent care HIV testing and linkage procedures are similar.

### Study Protocol

We used the hospital HIV clinic, Ward 86, to recruit individuals whose HIV diagnosis occurred in the ED or urgent care (UC) at least six months prior for in-person interviews in English or Spanish lasting one to one and a half hours. This time frame was chosen so as to be sensitive to individuals adjusting to living with HIV prior to recruiting them to talk about their HIV diagnosis in a research setting, especially since many individuals diagnosed with ED/UC in our hospital have pressing medical issues. We also wanted participants to be able to reflect on their linkage experiences. Recruitment was accomplished by using linkage team logs to identify patients diagnosed in the ED/UC and asking the providers of those patients to refer to our study; in addition, we posted flyers throughout the clinic asking potential participants to call a study number, upon which eligibility was confirmed through brief electronic medical record review.

The study team consisted of HIV physicians and nurses trained in qualitative methods, a medical sociologist, a medical anthropologist, and a clinical psychologist. The team developed an interview guide focused on the steps of the HIV care cascade [12] that was revised in an iterative fashion during the first several interviews to ensure yield and flow of information. After obtaining verbal consent, a medical sociologist or a nurse trained in qualitative methods (neither of whom was involved in the clinical care of the patients) conducted the interview in a private room in research space affiliated with the clinic. Interviews were audio-recorded and lasted about an hour. Participants were reimbursed $30. Interviewers wrote field notes after the interviews. Interviews were transcribed (and if in Spanish, translated) verbatim. Study data were collected from February 2011 to October 2012 until the study team felt the data had reached “saturation,” [13] or, the point where no new themes were being uncovered.

### Data Analysis

Data analysis focused on the sections of the transcripts from the parent study that described the HIV diagnosis and linkage to care experience. These experiences were elucidated by asking the following open-ended questions: “What brought you to the ED/UC on the day you were diagnosed with HIV?” “Can you walk me through what happened when you got your positive test result?” “How did you feel after you got your positive test result?” “Tell me about how you came to see a doctor or nurse for HIV care after leaving the hospital.” Coding for the parent study involved approaches that were both deductive, in which a conceptual framework from literature review helps to guide analysis, and inductive, in which codes arise from close reading and comparisons of text, consistent with grounded theory [14,15]. Two coders independently coded five transcripts and discussed discrepancies until >90% agreement was reached. Additional differences were minimal and resolved by consensus. Atlas. ti was used to code the data. The queried data for the code “diagnosis experience” was read aloud across interviews by four members of the interdisciplinary study team in a series of analysis meetings. Notes taken during these analytic meetings allowed the development and refinement of new codes, which were collated into themes.

## Results

### Participant Characteristics

Of all participants (n=24), fourteen were first diagnosed with HIV in the ED and ten were first diagnosed with HIV in the UCC. The median time since diagnosis was 24 months (range 6-62 months). The median age was 45 (range 25 ,61). The majority of participants were male and approximately two –thirds were from racial/ethnic minority groups (Table S1). At the time of HIV diagnosis, one-third had a psychiatric diagnosis, 50% reported using illicit substances, 80% did not have health insurance, and 62% were stably housed. One-half of participants were admitted to the hospital after the ED/UC visit that diagnosed them with HIV infection.

### Key Themes

Key diagnosis experience themes (Table S2) included physical discomfort and limited functionality (the ability to perform daily tasks), the presence of comorbid diagnoses, a wide spectrum of HIV risk perception, and feelings of isolation and anxiety. Nearly all participants described compassionate disclosure of test results by health care providers, although several noted logistical issues that complicated the disclosure experience. Key linkage to care themes (Table S2) included the importance of continuity between the testing site and the HIV clinic, hospital admission as an opportunity for support and education, and thoughtful matching by linkage staff to a primary care provider.

### Impact of Physical Discomfort, Limited Functionality and Co-Morbid Diagnoses

Participants described seeking care in the ED or UC because they felt very sick or had limited functionality for which they had no explanation. Indeed, feeling extremely ill led participants to accept the HIV test despite concerns, e.g., being undocumented or lacking health insurance, that might have prevented them from accepting the test in other settings. Many participants stated that a desire to feel physically better was the immediate focus of the HIV diagnosis experience.

I was so sick and I was so dehydrated that I wasn’t really (pauses) – all I wanted was someone to take care of me and make me feel better, you know, give me some fluids.
  - 49-year-old white MSM

When patients presented with urgent clinical conditions, providers often focused on stabilizing that condition. For example, one participant who presented with gastrointestinal bleeding and a very low blood count remembered clinicians prioritizing a blood transfusion.

When I thought about it to me being diagnosed with HIV is pretty serious but it just so happened at the same time I was diagnosed with all this other stuff. So it was just the way I was diagnosed. It was basically, “Oh, by the way you have HIV.” Like a checklist and stuff. I didn’t think much about it. I was shocked but they were focusing on everything else that was wrong: the colitis, the stomach problems, internal bleeding.
  - 47-year-old white MSM

Some participants recalled being diagnosed with other serious medical conditions that required immediate and intensive treatment, which took precedence over the HIV diagnosis.

I think that my issue was not about taking care of my HIV – it was taking care of the cancer and trying to recuperate and expel all the chemicals that were being put in me. How can you eat when you feel so nauseous? How can you exercise if you can’t even walk? I think that even to this day I was psychologically more traumatized (laughs) about that than having and being infected with HIV.
  - 43-year-old Asian man

### A Wide Spectrum of HIV Risk Perception - Shock, Betrayal, and Guilt

Perceptions of HIV risk varied, including those who expected the diagnosis, those who had no theory regarding their infection, and those who reported feeling betrayed by their partners. Many participants described a state of shock that left them unable to process the diagnosis.

p: She said, “You came back HIV positive.” I don’t know – after that I was in shock. I ran out into the parking lot and screamed.
  I: You really didn’t hear anything else she said?
  p: No, all I know is that they made an appointment for me at Ward 86.
  -43-year-old Latino woman

Some participants described not being able to have any emotional response at all.

I couldn’t even cry. I was just in shock. Because mostly I never thought I was going to – get positive from someone that – you know, we’ve been together a long time.
  - 32-year-old Latino MSM

Even months later, the absence of an acceptable explanation for HIV infection continued to affect acceptance of HIV-positive status. One participant put forth her own explanation.

Because like I said I was in a relationship for seven years and him and I got tested every year. So when they told me I felt like it was basically a conspiracy or them trying to help someone or pre-diagnose… I thought they were testing stuff on me. I started reading my TB clinic file and I noticed I didn’t have TB but since the medicine for TB was working for me they kept prescribing it for me. So I felt like that with the HIV.
  - 27-year-old African-American woman

Like this participant, those who did not perceive themselves as being at risk for HIV infection were less likely to know other HIV-positive individuals and to be able to imagine themselves as a person with HIV infection or identify with an HIV-positive community, resulting in immediate concerns about HIV stigma. The participants who knew individuals living with HIV and could envision a non-stigmatized HIV identity appeared to have lower levels of anxiety about disclosure and stigma.

In addition, even participants who were aware of their HIV risk reported feelings of guilt and moral judgment. One MSM respondent stated, “I was so mad at myself because I knew better.”

### Anxiety and Isolation

Another circumstance specific to this testing context with the potential to result in anxiety about disclosure is that friends or family who accompany an individual to the ED/UC may not be the people to whom that individual wishes to disclose their HIV status, making confidentiality an immediate concern. While those who seek out HIV testing in the community may bring a friend or partner to the testing venue for emotional support, some of those diagnosed with HIV in the ED/UC were alone at the time of diagnosis. Individuals who were alone at the time of diagnosis stated that a supportive response from health care providers was especially important to them.

I was by myself in the emergency room so I just felt horrible and there was nobody there and you know it was really bad. You know, the nurse she was really nice and she showed a lot of empathy for me. She told me, “It’s going to be okay.”
  - 25-year-old Latino MSM

Medical conditions that require physical isolation, such as tuberculosis, may potentiate the sense of isolation. One participant described how her suspected tuberculosis meant she was alone while awaiting her test results.

I came with my mother but they separated us and they had me waiting by myself, which was getting me more hysterical because I was starting to have a high fever. I had a nurse come every 15 or 20 minutes and she started consoling me but wouldn’t tell me what was wrong. She kept saying, “Are you okay? Do you have somebody who could be here with you?” I’m like, “My mom is outside.”
  - 27-year-old African-American woman

### Compassionate Disclosure with Some Logistical Hurdles

Indeed, most participants described compassion and kindness of the part of health care providers when receiving their diagnosis.

When I was diagnosed I was throwin’ my innards out. It was green and brown and like clay. What the hell is this shit? This lady – I forget her name but I’ll never forget her face – she was like, “It’s bile.” And I go, “I’m not a bad person.” She goes, “That’s bile. You are not vile.” She smiled and it made a difference.
  - 51-year-old white male IDU

No participant mentioned being stigmatized by a health care provider. However, several participants pointed out logistical issues that made the disclosure experience more difficult for them. One participant highlighted how important it was for the ED/UC to verify and update patient contact information if positive test results were disclosed after the visit.

All they had was my emergency contact number from a while ago, which was my mother. So the next morning I get an urgent call form my mother saying, “I just got a call from the hospital. You definitely need to call them right now.” And of course she’s frantic, wondering what’s going on, and they wouldn’t tell her anything. I knew instantly why they were calling. But they wouldn’t tell me on the phone.
  - 48-year-old white MSM

A Spanish-speaking participant emphasized wanting to have access to a Spanish-speaking staff person at the time of disclosure.

I asked them to do the favor of bringing me a person that spoke Spanish. In general, they were not rude but they weren’t pleasant. It took about an hour for someone to arrive – very kind – I think the woman was Puerto Rican – and she came – I think from another area – and she told me, “Don’t worry, I am going to tell you everything that is happening to you.
  - 45-year-old Latino MSM

### Continuity between the ED/UC Testing Site and the HIV Clinic

Participants described the value of continuity between the testing site and the HIV clinic. The Spanish-speaking participant who asked for a Spanish-speaking staff person said that person told him, “I am going to send you to see someone who I know will give you the help you need,” and took him to a Spanish-speaking linkage team member in the HIV clinic the next day. The participant who was called back to the UC for disclosure went on to describe how he was walked directly from the UC to the HIV clinic, given a tour, and immediately set up with a social worker. Another participant stated that he formed positive impressions about the HIV clinic from the way he was received there after his diagnosis in the ED, noting strong support and communication on the part of clinic staff.

I was really impressed with the (ED) doctor – she was very kind. She let me know she had set up an appointment for me to go to Ward 86. When I walked in the door of Ward 86, I was just held – I was pretty stunned and they did a really good job of letting each other know I was newly diagnosed. It was a really good transition from when I found out from the doctor to going to Ward 86.
  - 33-year-old Latino MSM

### Hospital Admission as an Opportunity for HIV Education and Building Trust

As noted in many studies of the HIV diagnosis experience, the response to HIV diagnosis was often characterized by a fear of death. Patients stated that the HIV education they received from the linkage team while in the hospital played a critical role in reframing their perceptions of HIV and HIV treatment.

A (linkage team member) and the doctor, they told me, ‘You can have 20-30 years. It’s like diabetes. I go, ‘What?’ I thought it was a death sentence. I didn’t know. That’s my ignorance. And then when it was explained to me I go, ‘What? I’m 50 years old. 80? You’re not gonna keep me alive that long, are ya? (laughs) But you can if you want.”
  - 51-year-old white male IDU

In cases where participants initiated antiretroviral therapy during the hospital admission, they reported that education about the purpose of the medications, as well as their potential toxicities and side effects, was crucial.

Nine different medicines! I needed to know what this was for, what this was for, what that was for. If they could do that the first time they meet a patient and then before they get discharged they could rule back a lot of confusion and distrust. Because personally I was like, “I don’t want to take it because I don’t know what I am taking.” Information is what builds your trust.
  - 27-year-old African-American woman

### Thoughtful Matching to an HIV Provider

After being linked to the HIV clinic, participants had to be linked to a permanent provider. Many participants expressed appreciation for the thought linkage team members put into choosing that person.

When I was being assigned to a nurse practitioner, she said, “I know exactly who I want you assigned to.” She did a process. She didn’t say, “So and so needs one more patient or can handle it.” She thought, “I think these two would have a connection.”
  - 61-year-old white MSM

Participants voiced that they felt strong connections to linkage team members and that they valued the opinion of the linkage team in choosing a permanent provider.

## Discussion

We found several aspects of the ED/UC diagnosis experience that have important implications for clinicians working in these environments. Patients who came to the ED/UC expressed feeling extremely ill and HIV test acceptance was motivated by their desire to obtain relief from severe discomfort. A focus on other medical concerns, such as ameliorating severe symptoms or the concomitant receipt of another serious diagnosis, e.g., cancer, may result in delayed emotional processing of the HIV diagnosis because the severity of the other condition had to take precedence in the immediate treatment plan. Similar to qualitative studies of individuals diagnosed due to HIV/AIDS-related signs or because of HIV-positive partners, perceptions of HIV risk varied such that the diagnosis experience of respondents ranged from confirming prior suspicions to being extremely traumatic [16–18]. Feelings of betrayal, sadness, shame and guilt were common. Unlike those who specifically seek out HIV testing, individuals in the ED/UC may be completely unprepared for HIV diagnosis. This state of shock can lead to difficulty accepting one’s HIV-positive status or the seeking of alternative narratives e.g. being part of an experiment. Indeed, a study of women who were not anticipating an HIV diagnosis found that subsequent denial of HIV status was common [19]. These individuals may be less informed about HIV and lack psychological strategies for handling fears of HIV stigma. The lack of a HIV risk identity can mean that individuals are less likely to be able to see themselves as a person with HIV and identify with an imagined or real HIV community. Acknowledging these facets of the ED/UC diagnosis experience is important, since acceptance of HIV diagnosis – or at least, believing the possibility that one might have HIV – is a necessary step in linking to care.

In addition, the ED/UC diagnosis experience can mean that patients do not have their desired emotional supports with them, because they may be alone or with family members or friends to whom they do not want to immediately disclose. Other patients may have no one they can rely on for immediate support. A sense of isolation can be potentiated by the hospital experience, especially when patients are placed in physical isolation for medical reasons. Health care providers can play key roles in providing guidance, education, emotional support, and sensitivity during these moments, and providers should not assume that the friends or family present in the ED/UC are the people to whom the individual wishes to disclose.

Importantly, no participant described stigmatizing behaviors on the part of ED/UC providers at the time of disclosure. Indeed, most participants noted kindness, compassion, and seamless linkage to the HIV clinic. “Active” linkage to care, in which testing or clinic staff members walk patients to the HIV clinic for the first time or patients meet a clinic staff member in the ED/UC, is an effective and increasingly popular method of linkage [20]. Indeed, the idea of a “first responder” to guide a patient through the diagnosis and linkage experience is not new [19]. Many of our participants remarked on active linkage in an appreciative way. These disclosure and linkage experiences appeared to help participants develop favorable attitudes toward the HIV clinic and its staff. For those patients who were admitted after their ED/UC diagnosis, inpatient hospitalization and the desire to get well provided the opportunity for education about HIV in general and antiretroviral drugs in particular. This education was important in building trust in the medical process and instilling a sense of hope about the future, as response to HIV diagnosis remains characterized by a fear of death, even thirty years into the epidemic. Finally, when linking to a permanent HIV provider, participants appreciated thoughtful matching on the part of linkage staff. Indeed, patients connected strongly to linkage staff and these relationships were instrumental in familiarizing patients with the HIV clinic and the initiation of HIV care.

Limitations of this study are that it was conducted at a single site, which may limit its generalizability, and that participants were recruited from the hospital HIV clinic. Different perceptions on the diagnosis experience may have been obtained from participants who never successfully linked to care or linked to care elsewhere, but it would be very difficult to recruit these participants for research. In addition, participants were interviewed at least six months after their HIV diagnosis. However, HIV diagnosis is a powerful event and participants appeared to have no trouble recalling their perceptions at that time. Finally, reflecting the nature of the HIV epidemic in San Francisco, the predominant risk group in this study was men who have sex with men. Our interviews with women and heterosexual men suggest that participants in these groups knew fewer HIV-positive individuals and had higher levels of internalized HIV stigma, but it possible that additional interviews may have identified other challenges.

## Conclusions

ED/UC testing programs and HIV providers should be sensitive to the unique roles of sickness, risk perception, and isolation in the ED/UC diagnosis experience, as these things may delay acceptance of HIV diagnosis, which is necessary for engagement in care. The disclosure and linkage to care experience is crucial in forming patient attitudes towards HIV and HIV care, thus staff involved in disclosure and linkage activities should be trained to deliver compassionate, informed, and thoughtful care that bridges HIV testing and treatment sites.

## Supporting Information

Table S1 Participant Characteristics. (DOCX)

Table S2 Key Themes in the Emergency Department/Urgent Care HIV Diagnosis and Linkage to Care Experience. (DOCX)
