# Supplementary material for: Patient Perspectives on the Experience of Being Newly Diagnosed with HIV in the Emergency Department/Urgent Care Clinic of a Public Hospital
Source: PLoS One. 2013 Aug 26;8(8):e74199. doi: 10.1371/journal.pone.0074199 (PMC3753265; doi:10.1371/journal.pone.0074199)
Supplement: Table S1 — (DOCX) [file pone.0074199.s001.docx]

**Table S1: Participant Characteristics (n=24)**

| **Category** | **Sub-Category** | **Result** |
| --- | --- | --- |
| Age | (median, range) | 45 (25-61) |
| Gender | Male | 20 (83%) |
|  | Female | 3 (13%) |
|  | Transgender (Male-to-Female) | 1 (4%) |
| Race/Ethnicity | White | 9 (37%) |
|  | African-American | 4 (17%) |
|  | Hispanic | 8 (33%) |
|  | Asian-American | 3 (13%) |
| HIV Risk Factor | Men Who Have Sex with Men (MSM) | 13 (55%) |
|  | Heterosexual | 7 (29%) |
|  | Intravenous Drug Use (IDU) | 2 (8%) |
|  | Not Sure | 2 (8%) |
| Insured at HIV Diagnosis |  | 5(21%) |
| Stably Housed at HIV Diagnosis |  | 15 (62%) |
| Prior Psychiatric Diagnosis |  | 9 (37%) |
| Using Illicit Substances at HIV Diagnosis* |  | 12 (50%) |
| Admitted After HIV Diagnosis |  | 12 (50%) |
| Time Since HIV Diagnosis | (median, range) | 2 years (6 months – 5 years) |

*Illicit substances include cocaine, crack, heroin, methamphetamines, speed, uppers, poppers, and marijuana.
