# Supplementary material for: Patient Perspectives on the Experience of Being Newly Diagnosed with HIV in the Emergency Department/Urgent Care Clinic of a Public Hospital
Source: PLoS One. 2013 Aug 26;8(8):e74199. doi: 10.1371/journal.pone.0074199 (PMC3753265; doi:10.1371/journal.pone.0074199)
Supplement: Table S2 — (DOCX) [file pone.0074199.s002.docx]

**Table S2: Key Themes in the Emergency Department/Urgent Care HIV Diagnosis and Linkage to Care Experience**

| **Thematic Category** | **Key Themes** | **Corresponding Challenge for Testing Programs** |
| --- | --- | --- |
| HIV Diagnosis | Physical Discomfort/Limited Functionality and Co-Morbid Diagnoses | Focus on other physical issues can lead to delayed processing of HIV diagnosis |
|  | Wide Spectrum of Risk Perception | Feelings of shock, shame, and betrayal can lead to denial of HIV status or prolonged time to acceptance of HIV status |
|  | Feelings of Isolation and Anxiety | Friends/family/partners with patient at diagnosis may not be the ones to whom that individual wishes to disclose |
|  |  | Importance of Provider Compassion |
| Linkage to Care | Importance of Continuity Between Testing and Care Site | Appropriate personnel to “bridge the gap” between the testing site and the care site |
|  | Hospital Admission as an Opportunity for HIV Education | Providing education and building trust |
|  | Thoughtful Matching to a Primary Care Provider | Experienced linkage staff familiar with HIV primary care providers |
